# Ultrasound-guided resection of cerebellar racemose neurocysticercosis: novel insights from a unique scenario

**DOI:** 10.1093/jscr/rjae249

**Published:** 2024-04-24

**Authors:** Rolando V Rojas-Apaza, Jhon E Bocanegra-Becerra, Henry Ruiz-Garcia, Jorge Rabanal-Palacios, Francisco Zambrano-Reyna

**Affiliations:** Department of Neurosurgery, Hospital Nacional Edgardo Rebagliati Martins, EsSalud, Lima, Peru; Academic Department of Surgery, School of Medicine, Universidad Peruana Cayetano Heredia, Lima, Peru; Department of Neurosurgery, University of Iowa, IA, United States; Department of Neurosurgery, Hospital Nacional Edgardo Rebagliati Martins, EsSalud, Lima, Peru; Department of Neurosurgery, Hospital Nacional Edgardo Rebagliati Martins, EsSalud, Lima, Peru

**Keywords:** racemose neurocysticercosis, Taenia solium, cerebellum, surgery, ultrasound, case report

## Abstract

Racemose neurocysticercosis (RNC) is a malignant form of *Taenia solium* infection. It carries high mortality due to widespread intraparenchymal invasion, mass effect, and cyst rupture. Cerebellar RNC is unusual and constitutes a surgical challenge. Scarce applications of ultrasound (US) -guided resection have been reported for RNC of the posterior fossa. We report the case of a 66-year-old woman who presented with ataxia and dysmetria. Her past medical history was relevant for seizures and hydrocephalus secondary to neurocysticercosis. Because of the increasing cyst invasion and threatening mass effect in the posterior fossa, the patient underwent US-guided resection of lesions. Postoperative computed tomography (CT) demonstrated complete excision of cysts, and a 2-year follow-up magnetic resonance imaging (MRI) showed no recurrence. On neurological examination, the patient had persistent ataxia without new-onset neurological deficits. The present case study illustrates the feasibility and cost-effective approach of US-guided resection to provide enhanced operative visualization and achieve complete cyst resection.

## Introduction

Racemose neurocysticercosis (RNC) is a malignant form of *Taenia solium* infection [1]. Parasite invasion of the cerebellar parenchyma has rarely been reported, yet it can be associated with high morbidity and mortality [2–4]. Remarkably, surgical resection of RNC is demanding because of its widespread location and compromise of vital structures [1]. For this reason, diligent execution and holistic intraoperative visualization of the affected tissue are paramount. Over the last few decades, ultrasound (US) has represented an important tool to assist in cerebral lesion resection, owing to its potential to provide real-time visualization at different surface planes [5–8]. Herein, we aim to illustrate the feasibility and utility of performing US-guided lesion resection for cerebellar RNC.

## Illustrative case

**Figure 1:**
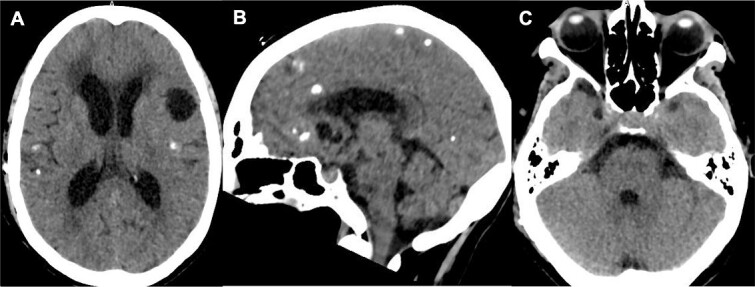
Initial brain CT revealed multiple calcified cysts with ventriculomegaly in the axial (A, C) and sagittal (B) views.

**Figure 2:**
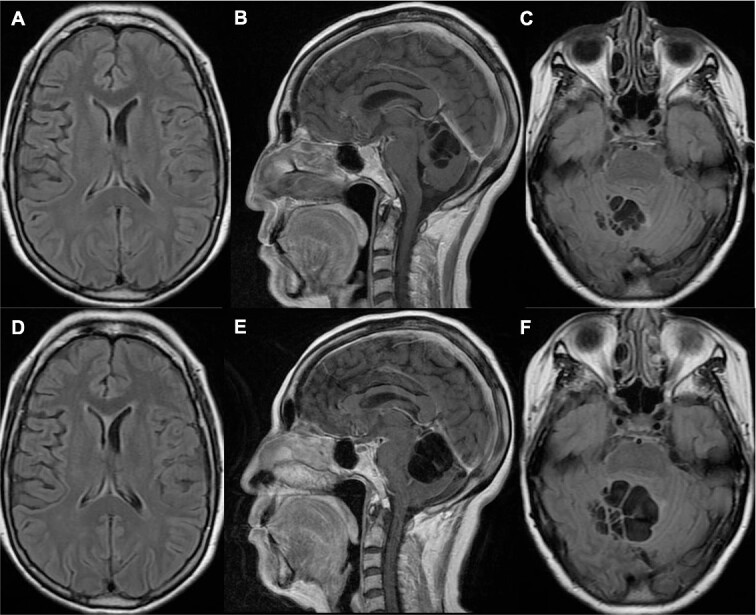
Brain MRI one year (A–C) and 14 months (D–F) prior to her current admission. (A–B) T1 sequence depicted multiple cystic lesions in the cerebellum without significant compression of the fourth ventricle; (C) T2 fluid-attenuated inversion recovery (FLAIR) sequence showed slight perilesional edema; (D–E) T1 sequence demonstrated a notable increase in cyst growth along with compression of the fourth ventricle; (F) T2 FLAIR sequence showed increased edema.

A 66-year-old woman presented with ataxia and dysmetria. She had a past medical history of hydrocephalus and seizures secondary to neurocysticercosis that merited medical management with a 30-day protocol of albendazole administration at 15 mg/kg/d and placement of a ventriculoperitoneal shunt (Fig. 1). Over the year before admission, brain imaging revealed multiple and increasing cystic lesions in the right cerebellar hemisphere (Fig. 2A–C). Besides, a follow-up brain MRI with contrast revealed multiple contrast-enhancing cysts compressing the fourth ventricle, perilesional edema, and an extra ventricular component next to the cerebellar tentorium (Fig. 2D–F).

Upon evaluating the patient’s clinical status and the imminent risk of herniation as a result of the cystic mass effect in the posterior fossa compartment, she was deemed a candidate for microsurgical resection.

### Operative note

**Figure 3:**
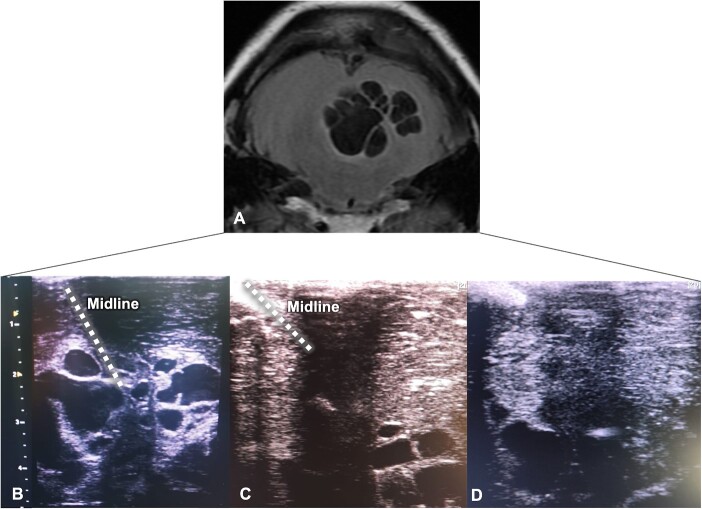
Intraoperative ultrasound-guided resection. (A) Cerebellar MRI is shown upside down for better correlation with the ultrasound view; parasitic cysts correspond to hypointense lesions; (B–D) Echographic views depicted the progression of ultra-sound guided resection with excision of the hypoechoic lesions from the superficial to deep planes.

**Figure 4:**
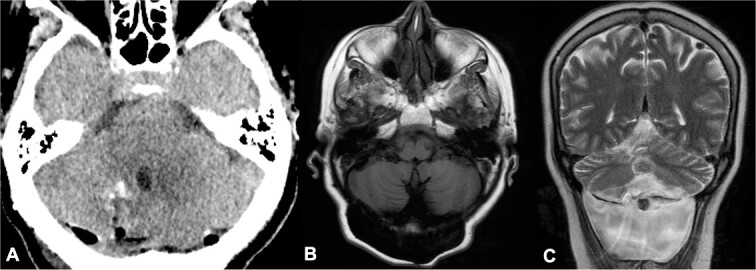
Postoperative head CT and 2-year follow-up brain MRI. (A) Axial view of head CT demonstrated complete resection of cysts; (B–C) 2-year follow-up brain MRI showed no recurrence of parasitic cysts in T1 axial and T2 coronal sequences and a cerebrospinal fluid fistula.

The patient was placed in a Concorde position, and a standard sub-occipital craniotomy was performed. Drilling of the occipital rim and a double Y-shaped durotomy ensured greater visualization of the supra-cerebellar infratentorial corridor. Subsequently, the resection of parasitic lesions was assisted by utilizing a 2D ultrasound linear transducer (Samsung® Medison Sonoace X6). By correlating the cyst depth locations in the MRI, the transducer provided visualization of lesions from the most profound plane (located at 4 cm) up to the surface (Fig. 3A and B). Mobilization of the transducer in acute angles from the midline increased the angle of attack for the complete resection of lesions (Fig. 3B–D). Lesions were sent for histopathological examination, confirming the diagnosis of *Taenia solium* cysts*.* A postoperative CT without contrast corroborated the complete resection of cysts (Fig. 4A).

On neurological examination, ataxia persisted without new-onset neurological deficits. A recent 2-year follow-up brain MRI showed no recurrence of cyst formation and a cerebrospinal fluid fistula, which is currently awaiting surgical management (Figs. 4B and C).

## Discussion

### Racemose neurocysticercosis of the cerebellum

RNC lesions typically have a cystic appearance without a scolex in the parenchyma, ventricles, or extra ventricular tissue [1, 3]. Invasion of the subarachnoid space or parenchymal tissue is less frequent but has a greater risk of cystic growth, given the expansible nature of these compartments [3]. As a result, patients can develop clinical symptoms and signs secondary to space-occupying lesions, such as obstructive hydrocephalus, meningitis, and potentially herniation, with a mortality rate of up to 50% [2, 4]. In addition, RNC with cerebellar involvement has scarcely been reported [2, 9, 10].

Medical management for parenchymal neurocysticercosis has included dual therapy with Albendazole (15 mg/kg/day) and Praziquantel (50 mg/kg/day), when two or more viable cystic lesions are present on imaging [4]. Nevertheless, surgical excision of neurocysticercosis lesions can be necessary, especially in scenarios where medical therapy has failed or there is an imminent risk of parenchymal herniation [2, 11]. Surgery demands careful preoperative planning and exquisite recognition of the cystic anatomy intraoperatively to provide safe resection. Furthermore, the number of cysts and the presence of adherent gliotic tissue are noteworthy factors to consider for a gentle execution.

### Application of ultrasound-guided resection in neurosurgery

Since 1982, the utilization of the US in brain surgery has gained significant attention to guide lesion resection [6]. Subsequent developments in ultrasound technology, such as three-dimensional and contrast–enhanced US, have improved the quality of visualization with the possibility of studying deeper anatomical structures [5–8]. The utility of US lies in the straightforward identification of normal and pathological structures according to their echogenicity [6]. For example, the sulci, falx cerebri, choroid plexus, and vessel walls appear hyperechoic. Hypoechoic regions (i.e. acoustically homogeneous) include the ventricles and cisterns filled with cerebrospinal fluid [6]. The brain parenchyma is relatively uniform, with gray matter appearing slightly hyperechoic relative to white matter. On the other hand, most tumors appear hyperechoic as a result of their relatively high mass density, and acute edema and cystic lesions appear hypoechoic [6].

Major applications of US-guided resection include excision of high-grade gliomas and metastasis [7]. On the other hand, tenets for safe resection of posterior fossa lesions have been scarce. Šteňo *et al.* [10] reported the resection of a cerebellar pilocytic astrocytoma guided by three-dimensional-US. Important insights from this case included the preference for the prone position over the Park-bench position to fill the resection cavity with saline for better US visualization [10]. Additionally, consideration of entrapped air is necessary because it may cause acoustic artifacts and appear as a false empty resection cavity [8].

### Ultrasound-guided resection of racemose neurocysticercosis

Resection of extra ventricular RNC of the cerebellum is a demanding task. Notable technical efforts to achieve delicate excision have included the use of a stereotactic frame to guide the resection of the fourth ventricle cysticerci [11], and the use of a retrosigmoid approach to gain better visualization of the extension of cysts into the cisterns [10].

To the best of our knowledge, we report for the first time the use of US-guided resection for a cerebellar RNC lesion. In this case study, guidance from the US was paramount to observe the localization of cysts in real time and achieve complete excision across all planes. Besides, the clear delineation of a resection plane was facilitated by the echogenic contrast between the cyst (hypoechogenic) and cerebellar parenchyma (isoechogenic). Moreover, adequate filling of the resection cavity with saline was relevant to avoid air entrapment and acoustic interference.

### Advantages and limitations of ultrasound-guided resection

The low cost, ease of use, and real-time feedback have made US-guided resection an appealing tool for the excision of brain lesions, especially in settings where intraoperative MRI may not be available [5]. Nevertheless, it has several limitations worth acknowledging: (i) US resolution is not uniform across all transducer directions and relies heavily on the depth of the area of interest, (ii) accuracy of lesion’s location can be affected by the presence of saline and the depth of the lesion relative to the transducer, (iii) it is an operator-dependent technique, and (iv) interpretation across modalities of imaging can be challenging because the rendering of US is given in a perpendicular plane to the transducer, which difficults the correlation with the normal view on CT or MRI [6–8, 10].

## Conclusion

Cerebellar RNC is a rare and burdensome entity. Surgical excision of parasitic cysts constitutes a lifesaving approach and demands careful operative planning for holistic visualization of lesions and gentle execution. 

The present case study provides meaningful insights from the pioneered use of US-guided resection of RNC lesions in the cerebellum. Significant advantages of US include improved real-time visualization of cyst lesions and assistance in delimiting the plane of resection.

## Conflict of interest statement

None declared.

## Funding

None declared.
